# Draft genome sequence of *Yoonia* sp. strain MH D7 isolated from the Antarctic green macroalga *Monostroma hariotii*

**DOI:** 10.1128/mra.00045-26

**Published:** 2026-04-20

**Authors:** Waldo Acevedo, Edith González-Ramos, Angara Zambrano, Evelyn Donoso, Francisco J. Morera, Luis Vargas-Chacoff, Sergio Leiva Poveda

**Affiliations:** 1 Faculty of Sciences, Institute of Chemistry, Pontificia Universidad Católica de Valparaíso 28047, Valparaíso, Chile; 2 Center for Interdisciplinary Research in Biomedicine, Biotechnology and Well-Being (CID3B), Pontificia Universidad Católica de Valparaíso 28047, Valparaíso, Chile; 3 Faculty of Sciences, Institute of Biochemistry and Microbiology, Universidad Austral de Chile 28040, Valdivia, Chile; 4 School of Veterinary Medicine, Pontificia Universidad Católica de Chile 28033, Santiago, Chile; 5 Faculty of Sciences, Institute of Marine and Limnological Sciences, Universidad Austral de Chile 28040, Valdivia, Chile; 6 Millennium Institute Biodiversity of Antarctic and Sub-Antarctic Ecosystems (BASE), Universidad Austral de Chile 28040, Valdivia, Chile; California State University San Marcos 14673, California, USA

**Keywords:** *Yoonia*, *Monostroma hariotii*, Antarctica, draft genome, macroalgae

## Abstract

We report the draft genome sequence of *Yoonia* sp. MH D7, an Alphaproteobacteria isolated from the surface of the green seaweed *Monostroma hariotii* collected in King George Island, Antarctica. The genome assembly comprised 3,862,164 bp, with a G + C content of 54.8% and 3,934 coding sequences.

## ANNOUNCEMENT

A *Yoonia* strain, MH D7, was isolated from the Antarctic green macroalga *Monostroma hariotii*. Members of the *Yoonia–Loktanella* clade are mainly reported from marine environments, including seawater (1), sediment (2), and the phycosphere of algal species (3–6). Reports from Antarctic habitats are scarce, with examples including microbial mats in Antarctic lakes (7), marine sponges (8), and bacterioplankton in the southern Weddell Sea (9), underscoring the value of sequencing the MH D7 genome.

MH D7 was isolated from the surface of healthy *M. hariotii* fronds collected from the intertidal zone at Rodríguez Point (62°11′57″ S, 58°56′34″ W), King George Island, Antarctica, in February 2014. Samples were placed in sterile plastic bags, transported at 4°C to the “Base Prof. Julio Escudero” laboratory on King George Island, and processed within 3 h (10). Fronds were rinsed three times with filtered sterile seawater (FSSW), cut into small pieces, subjected to serial dilution in FSSW, and plated on Marine Agar 2216 (BD). Plates were incubated at 4 and 20°C for up to 4 weeks and inspected regularly. Colonies were purified by repeated streaking, and purity was confirmed by Gram staining and colony morphology.

Genomic DNA was extracted from 72 h Marine Broth 2216 cultures using the GeneJET Genomic DNA Purification Kit and quantified using a Qubit fluorometer. Whole-genome sequencing was performed at AUSTRAL omics (Valdivia, Chile). Libraries were prepared from 400 ng DNA with the ONT Native Barcoding Kit 24 V14 and sequenced on a MinION using a Flongle (R10.4.1) flow cell. Bead-based clean-ups followed the ONT protocol, without additional size selection. Basecalling was performed using Dorado v0.5.1 (high-accuracy model), and read quality was assessed with NanoPlot v1.42.0 (11); reads with *Q* < 7 were discarded. The genome was assembled *de novo* using Flye (v2.9.1) and annotated through PROKKA (v1.14.6) (12) and the NCBI PGAP v6.10. To identify enzymes involved in carbohydrate metabolism, an HMMER (v3.3) search was performed against the CAZyme database via the dbCAN3 server (*E* value < 1.0 × 10⁻¹⁵, coverage > 0.35) (13). ANIb was calculated using JSpeciesWS (v5.0.3) (14), and dDDH was estimated with TYGS (v.2019.05.19) (15). Default parameters were used unless otherwise specified.

The full 16S rRNA gene was extracted from the whole-genome sequence. The EzBioCloud 16S database v.2025.04.21 (https://www.ezbiocloud.net/) (16) was used for sequence comparison. Based on pairwise 16S rRNA gene similarity, MH D7 showed the highest similarity to *Yoonia algicola* G8-12^T^ (96.09%; ON527811) and *Yoonia tamlensis* DSM 26879^T^ (96.08%; DQ533556). At the whole-genome level, MH D7 was closest to *Y. tamlensis* DSM 26879ᵀ (GCF_900115105.1; 71.70% ANIb), while dDDH (formula 4) gave a maximum of 20.3% to *Y. ponticola* DSM 101064ᵀ (GCF_014199395.1), both well below species thresholds, indicating MH D7 likely represents a distinct, previously undescribed lineage within *Yoonia*.

Oxford Nanopore sequencing yielded 74,891 reads (mean 5.9 kbp, median 3.2 kbp; mean quality 11.6, median 13.1), totaling 442.9 Mb, with a raw read *N*_50_ of 11,629 bp. The MH D7 genome assembled into five contigs (*N*_50_ 3.3 Mb; 9,075–3.29 Mb) with 99.41% completeness and ~108× coverage. The 3,862,164 bp genome (54.8% G + C content) encodes 3,934 coding sequences and 55 RNA genes (9 rRNA, 45 tRNA, and 1 tmRNA). The genome encodes diverse sugar- and polysaccharide-metabolizing proteins, including enzymes that degrade cellulose-derived oligosaccharides (GH1 and GH3) and putative chitin-degrading enzymes (GH18 and GH23), suggesting that *Yoonia* sp. MH D7 contributes to the degradation and recycling of algal-derived organic matter.

## Data Availability

All data are available under BioProject PRJNA1391519. Raw reads are deposited as accession SRR36760490, the genome assembly under GenBank accession JBTJCF000000000, and DRAM annotations at https://doi.org/10.6084/m9.figshare.31405374. This is the first version of the genome.
